# Mid-term outcomes associated with thoracic endovascular aortic repair and differences in outcomes for thoracic aortic aneurysms versus type B aortic dissections

**DOI:** 10.34172/jcvtr.026.33435

**Published:** 2026-03-30

**Authors:** Taiwo Dodo-Williams, Daniel Willie-Permor, Shima Rahgozar, Isaac Naazie, Kevin Yei, Mahmoud B. Malas

**Affiliations:** ^1^Division of Vascular and Endovascular Surgery, Department of Surgery, University of California San Diego, California, USA; ^2^Division of Vascular and Endovascular Surgery, Department of Surgery, University at Buffalo, Buffalo, USA

**Keywords:** Thoracic endovascular repair, Thoracic aortic aneurysm, Type B aortic dissection

## Abstract

**Introduction:**

Thoracic endovascular aortic repair (TEVAR) has become the preferred treatment modality of thoracic aortic pathologies. Since survival outcomes are influenced by disease acuity and pathology, we aim to compare mid-term outcomes of TEVAR in the management of thoracic aortic aneurysms (TAA) vs. Stanford type-B aortic dissections (TBAD).

**Methods:**

Patients undergoing TEVAR (2010-2019) in the Vascular Quality Initiative-Medicare-linked database were included in this analysis. Patients were divided into two groups: TAA and TBAD. Postoperative outcomes included 30-day mortality, stroke, myocardial infarction (MI), and spinal cord ischemia (SCI). Mid-term outcomes included 1 and 2-year all-cause mortality, reintervention and rupture.

**Results:**

Of the 2105 patients undergoing TEVAR in this analysis, 1,492 (70.9%) had TAA while 613 (29.1%) had TBAD. The TBAD group had a significantly lower 30-day mortality (aOR:0.67,95%Cl:0.46-0.98,*P*=0.037), lower 1-year mortality (aHR:0.72,95%CI:0.57-0.90, *P*=0.004) and also at 2 years (aHR:0.79,95%CI:0.65-0.95, *P*=0.013). Within 1 year, patients with TBAD had a significantly higher incidence of aneurysm related reintervention (18.8% vs. 12.9%, *P*=0.003). This finding persisted at 2 years (24.9% vs. 19.7%, *P*<0.001). There was no significant difference in rupture between groups at 2 years (4.1% vs. 3.4%, *P*=0.40).

**Conclusion:**

In this large multi-institutional study, patients undergoing TEVAR for TBAD had significantly lower postoperative mortality but a higher risk of reintervention at one and two years compared to patients with TAA. Additionally, both groups demonstrated a similar risk of rupture at both 1 and 2 years post-TEVAR. The rate of post-TEVAR rupture in both groups underscore the need for careful follow-up and reintervention as needed to maintain the integrity of the repair.

## Introduction

 Since its U.S. Food and Drug Administration (FDA) approval in 2005^1^, thoracic endovascular aneurysm repair (TEVAR) has become the preferred treatment modality of thoracic aortic pathologies due to a significant reduction in perioperative morbidity and mortality, incidence of spinal cord ischemia (SCI), hospital length of stay and promotion of aortic remodeling.^2-5^ Specifically, in patients with thoracic aortic aneurysms (TAA), the Gore Thoracic Aortic Graft (TAG) trial reports a lower 30-day mortality of 2.1% when compared to open repair 11.7%.^4^ In the management of acute aortic syndromes, such as aneurysms and dissections, endovascular aortic repair is associated with a lower 30-day mortality of 12.7% when compared to that of open repair which ranges between 25% to 45%.^6^ This has led to the current shift in management from open to endovascular repair.

 TAA repair rates have remained stable, while the repair rates of Stanford type B aortic dissections (TBAD) have increased over the years.^7^ This may be explained by current management guidelines for the different pathologies. The Society for Vascular Surgery currently recommends surgical repair for TAA when the aortic diameter reaches 5.5 cm or greater in asymptomatic patients but for patients with high risk of complications from TEVAR, a larger diameter threshold is proposed.^8^ TBAD is initially managed medically, with surgical repair reserved for aneurysmal expansion, organ malperfusion and failed medical management with refractory pain. Selected studies, however, suggest that earlier surgical intervention for some patients may be beneficial and improve long-term survival.^9,10^ High risk features in patients with dissections that warrant immediate intervention include pain and hypertension that is unresponsive to medical management, CT findings suggesting bloody effusion, aortic diameter greater than 40 mm, primary tear larger than 1 cm, and radiographic malperfusion.^11^

 Though survival outcomes are influenced by disease acuity and pathology, in this study, we aim to compare mid-term outcomes to assess the performance of TEVAR in the management of TAA and TBAD using the Society for Vascular Surgery Vascular Quality Initiative (VQI) registry. For more extensive mid-term outcomes such as mortality, rupture and reintervention, we will utilize the VQI Vascular Implant Surveillance and Interventional Outcome

 Network (VISION), a registry that links patients and procedures from the VQI dataset with Medicare claims data.

## Material and Methods

###  Database and Study Population

 Patients undergoing TEVAR between August 2010 and December 2019 in the SVS VQI VISION database were included in this analysis. The VQI includes data from over 1,000 centers across North America and contains de-identified data on major vascular procedures, including demographics, comorbidities, intraoperative details, and outcomes. To ensure data quality, the registry is subjected to regular, stringent auditing methods. More information on the VQI can be found at www.vqi.org. Since only deidentified data were used, the study was exempt from Institutional Review Board (IRB) review and individual patient consent. The study was approved by the SVS Data Safety Organization (protocol number: 4508).

 Patients were divided into two groups based on pathology: TAA and TBAD. To further characterize TAA, only patients with degenerative aneurysms were included. Patients with dissections and those with aneurysms resulting from dissection were included in the TBAD group. Pathology confined to the aortic arch (distal zone of disease < 3), thoracoabdominal aorta (distal zone of disease > 5), abdominal aorta (proximal zone of disease > 5), and dissections originating in zone 0 were excluded. Patients with traumatic aortic injury, penetrating ulcer, intramural hematoma, and aortic thrombus were also excluded.

 Baseline characteristics compared between the two groups included: age, gender, race, body mass index (BMI), symptomatic presentation, hypertension, coronary artery disease (CAD), congestive heart failure (CHF), chronic obstructive pulmonary disease (COPD), chronic kidney disease (CKD), dialysis, diabetes, stroke, prior aneurysm repair, prior carotid endarterectomy (CEA), carotid artery stenting (CAS), coronary artery bypass grafting (CABG), or percutaneous coronary intervention (PCI), prior bypass/peripheral vascular intervention (PVI), smoking status, preoperative medication use, preoperative maximum aortic diameter, American Society of Anesthesiologists (ASA) class, anesthesia type, and procedural urgency. Presentation was categorized into asymptomatic, symptomatic and rupture. HTN was defined as a documented blood pressure of ≥ 130/80 on 3 or more occasions. CAD was defined as a history of angina or myocardial infarction (MI). CKD was defined as an estimated glomerular filtration rate < 60 mL/min/1.73 m^2^. Smoking was divided into three categories: never, prior (quit ≥ 1 month ago) and current (still smoking within the last month). Urgency was divided into three categories: elective (scheduled procedure), urgent (surgery within 24 hours of presentation or acute presentation) and emergent (surgery within 4 hours of presentation or acute decompensation).

###  Outcomes

 Primary outcomes of interest included postoperative 30-day mortality, and in-hospital stroke, MI, and spinal cord ischemia (SCI). Secondary outcomes included 1 and 2-year all-cause mortality, reintervention, and rupture. Reintervention was defined as any new intervention related to the initial pathology, either aneurysm or dissection.

###  Statistical Analysis

 Continuous baseline characteristics were compared using a two-sample t-test, while categorical baseline characteristics were compared using Pearson χ2 test or Fisher’s exact test. Univariable and multivariable logistic regression analyses was used to assess postoperative outcomes, clustered by centers. Initial models included baseline characteristics. Variables from the resulting model were selected based on clinical relevance or backward stepwise selection with a threshold of *P* < 0.10 (Table 1).

**Table 1:** Demographics

**Patient characteristics**	**TAA (n=1492, 70.9%)**	**TBAD (n=613, 29.1 %)**	***P*** ** value**
Age, median (SD)	76.0(7.7)	71.9(9.6)	< 0.001
Gender			< 0.001
Male	731(49.0)	359(58.6)	
Female	761(51.0)	254(41.4)	
Race			< 0.001
White	1127(75.5)	420(68.5)	
Black	257(17.2)	143(23.3)	
Other	108(7.2)	50(8.2)	
BMI (kg/m^2^, SD)	27.2(6.4)	28.1(6.1)	< 0.001
Presentation			< 0.001
Asymptomatic	1006(67.5)	205(33.5)	
Symptomatic	363(24.3)	369(60.3)	
Rupture	122(8.2)	38(6.2)	
Hypertension	1370(92.1)	566(92.5)	0.75
CAD	326(21.9)	117(19.1)	0.15
CHF	228(15.3)	88(14.4)	0.58
COPD	591(39.6)	153(25.0)	< 0.001
CKD	4(0.3)	2(0.3)	0.82
Dialysis	48(3.2)	25(4.1)	0.33
Diabetes	266(17.8%)	98(16.0)	0.31
Stroke	220(14.8)	82(13.4)	0.41
Prior procedures			
Aneurysm repair	529(35.5)	159(25.9)	< 0.001
CEA/CAS	74(5.0)	11(1.8)	< 0.001
CABG/PCI	407(27.3)	106(17.3)	< 0.001
Lower extremity revascularization (Bypass/PVI)	196(13.2)	72(11.9)	0.39
Smoking			< 0.001
Never	307(20.6)	224(36.6)	
Prior	802(53.9)	267(43.6)	
Current	380(25.5)	121(19.8)	
Preoperative medications			
Aspirin	863(57.9)	299(48.9)	< 0.001
P2Y12 Antagonist	153(10.3)	31(5.1)	< 0.001
Statin	985(66.1)	350(57.3)	< 0.001
Beta blockers	1043(70.0)	478(78.2)	< 0.001
ACE-inhibitor/ARB	615(41.2)	276(45.2)	0.10
Anticoagulants	212(14.2)	106(17.3)	0.07
ASA class			< 0.001
I/II	29(1.9)	7(1.1)	
III	630(42.3)	165(26.9)	
IV/V	831(55.8)	441(71.9)	
General anesthesia	1438(96.4)	598(97.6)	0.38
Maximum aortic diameter (mm, SD)	58.9(13.7)	49.4(22.1)	< 0.001
Urgency			< 0.001
Elective	1195(80.1)	379(61.8)	
Urgent	174(11.7)	164(26.8)	
Emergent	123(8.2)	70(11.4)	

TEVAR: Thoracic endovascular aortic repair, TAA: Thoracic aortic aneurysm, TBAD: Type B aortic dissection, CAD: Coronary artery disease, CHF: Congestive heart failure, COPD: Chronic obstructive pulmonary disease, CKD: Chronic kidney disease, CEA: Carotid endarterectomy, CAS: Carotid artery stenting, CABG: Coronary artery bypass grafting, PCI: Percutaneous coronary intervention, ACE: Angiotensin-converting enzyme inhibitor, ARB: Angiotensin II receptor blocker, ASA: American Society of Anesthesiologists

 To compare mid-term outcomes, Kaplan-Meier analysis, log-rank tests, and Cox proportional hazards regression clustered by centers were utilized. Initial models included baseline characteristics. Variables from the resulting model were selected based on clinical relevance or backward stepwise selection with a threshold of *P* < 0.10. Hosmer-Lemeshow and Area Under the Curve (AUC) tests were used to assess the calibration and discrimination of the models. For logistic regression models, model discrimination and calibration were assessed using the Area Under the Curve (AUC) and Hosmer-Lemeshow goodness-of-fit tests. For Cox proportional hazards models, [e.g., Harrell’s C-index] was used to evaluate discrimination.

 All analysis was completed using StataSE version 16.0. A *P *value < 0.05 was considered statistically significant.

## Results

###  Demographic and Baseline Characteristics

 Of the 2105 patients undergoing TEVAR in this analysis, 1,492 (70.9%) had TAA while 613 (29.1%) had TBAD. On baseline characteristics, patients with TBAD were more likely to be male (58.6% vs.49.0%, *P* < 0.001), have a higher BMI (28.1 kg/m^2^ vs. 27.2 kg/m^2^, *P* < 0.001), be symptomatic (60.3% vs. 24.3%, *P* < 0.001), use beta-blockers preoperatively (78.2% vs. 70.0%, *P* < 0.001), belong to ASA class IV or V (71.9% vs. 55.8%, *P* < 0.001) and present urgently (26.8% vs. 11.7%, *P* < 0.001). Patients with TAA were more likely to be older (76.0 vs. 71.9, *P* < 0.001), white (75.5% vs. 68.5%, *P* < 0.006), actively smoking (25.5% vs. 19.8%, *P* < 0.001), have larger aortic diameter (58.9 mm vs. 49.4 mm *P* < 0.001), COPD (39.6% vs. 25.0%, *P* < 0.001), prior aneurysm repair (35.5% vs. 25.9%, *P* < 0.001), CEA/CAS (5.0% vs. 1.8%, *P* < 0.001) and CABG/PCI (27.3% vs. 17.3%, *P* < 0.001). In terms of preoperative medication, the TAA group had a higher usage of aspirin (57.9% vs. 48.9%, *P* < 0.001), P2Y12 inhibitors (10.3% vs. 5.1%, *P* < 0.001), and statins (66.1% vs. 57.3%, *P* < 0.001). (Table 1)

###  Postoperative Outcomes 

 The crude 30-day mortality rate was similar in TBAD compared to TAA (6.9% vs. 7.0%, *P* = 0.88). In the adjusted analysis, the TBAD group had a significantly lower 30-day mortality (aOR:0.67, 95%Cl: 0.46-0.98, *P* = 0.037).

 Patients with TBAD had similar rates of postoperative MI when compared to TAA (1.3% vs. 2.4%, *P* = 0.13, aOR:0.45, 95%Cl: 0.18-1.09, *P* = 0.08), post-op stroke (4.3% vs. 4.3%, *P* = 0.97, aOR:0.83, 95%Cl: 0.52-1.33, *P* = 0.44) or SCI (4.4% vs. 3.2%, *P* = 0.15, aOR:1.14, 95%Cl: 0.68-1.89, *P* = 0.62) (Table 2).

**Table 2:** Postoperative outcomes following TEVAR for TBAD vs. TAA

	**Univariable**			**Multivariable**	
**Outcome**	**TAA (n=1492, 70.9%)**	**TBAD (n=613, 29.1 %)**	***P*** **-value**	**Adjusted OR (95%CI) REF=TAA**	***P*** ** value**
30 Day Mortality	105 (7.0%)	42 (6.9%)	0.88	0.67 (0.46-0.98)	0.037
Stroke	64 (4.3%)	26 (4.3%)	0.97	0.83 (0.52-1.33)	0.44
MI	35 (2.4%)	8 (1.3%)	0.13	0.45 (0.18-1.09)	0.08
Spinal Cord Ischemia	47 (3.2%)	27 (4.4)	0.15	1.14 (0.68-1.89)	0.62

TAA: Thoracic aortic aneurysm, TBAD: Type B aortic dissection, OR: Odds ratio, CI: Confidence interval

###  Mid-Term Outcomes

**Figure 1:**
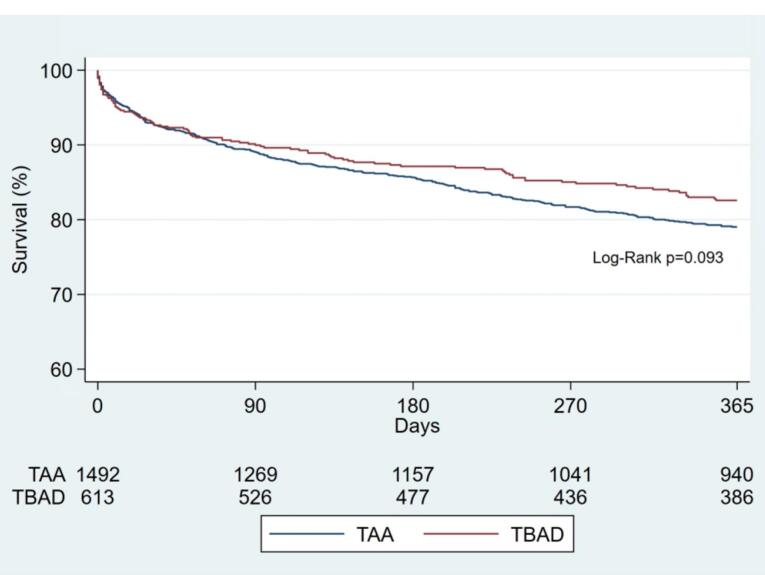
1-year All-cause Mortality by Pathology

**Figure 2:**
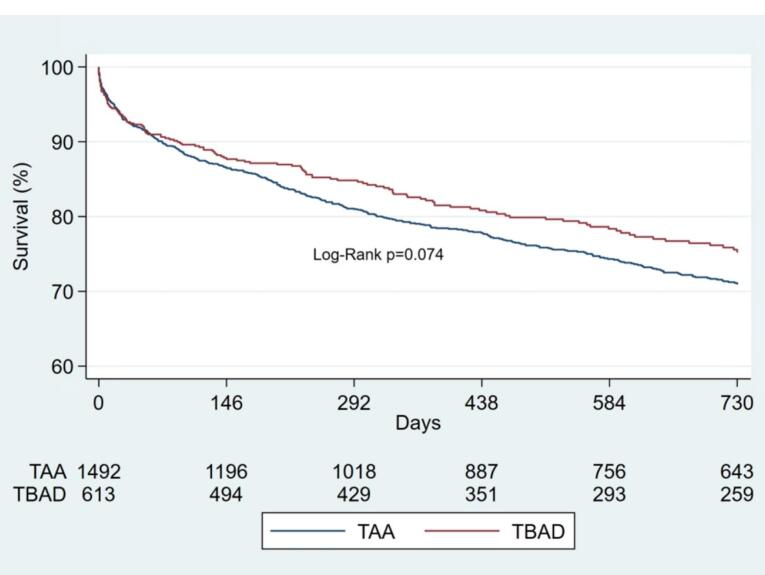
2-year All-cause Mortality by Pathology

**Figure 3:**
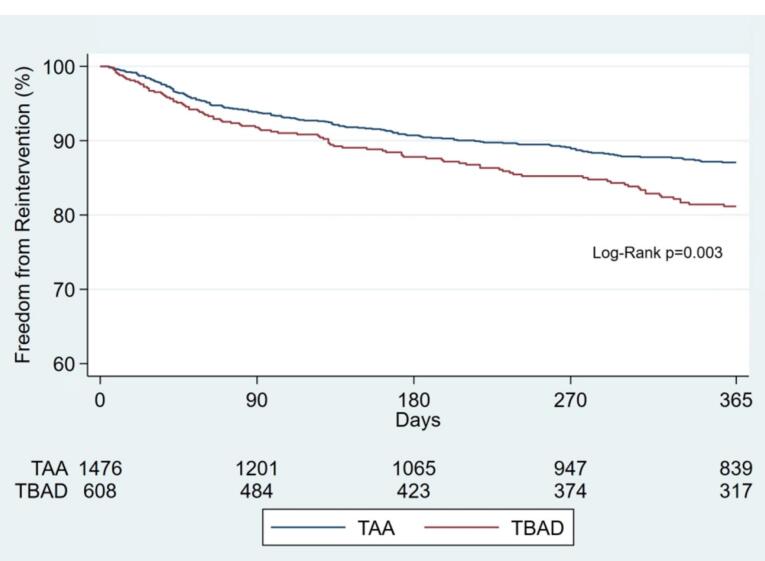
1-year Freedom from Reintervention by Pathology

**Figure 4:**
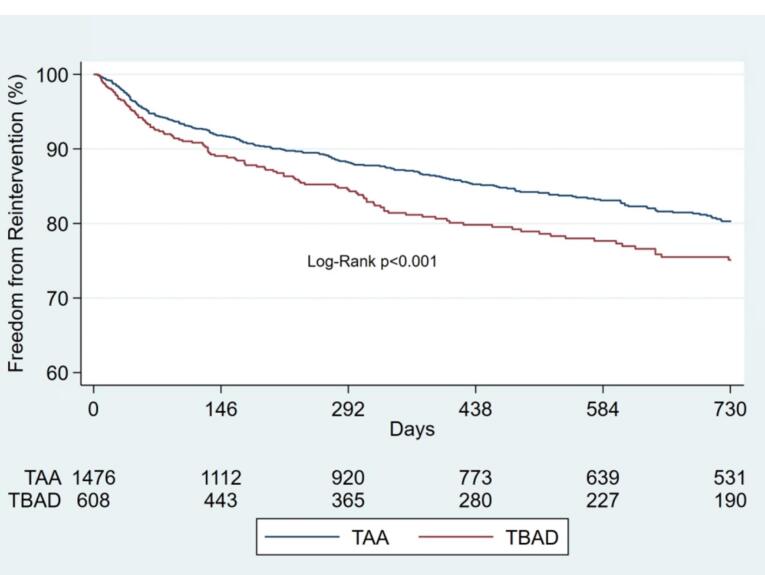
2-year Freedom from Reintervention by Pathology

**Figure 5:**
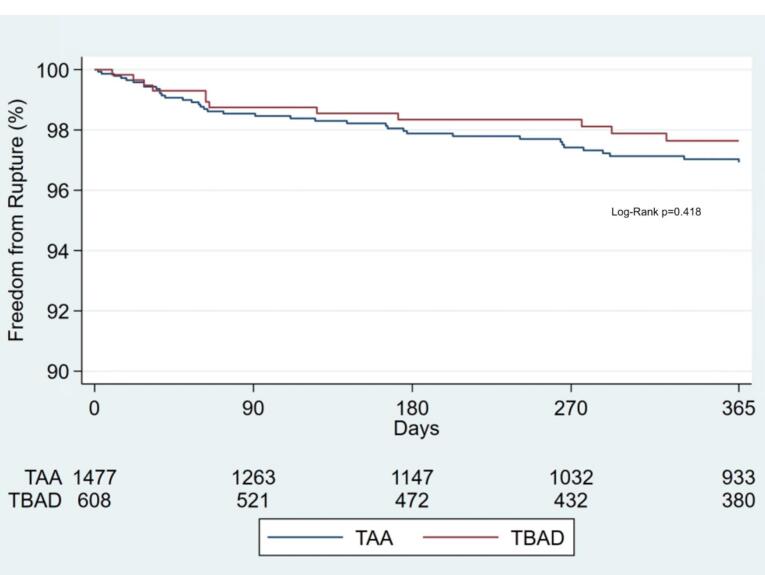
1-year Freedom from Rupture by Pathology

**Figure 6:**
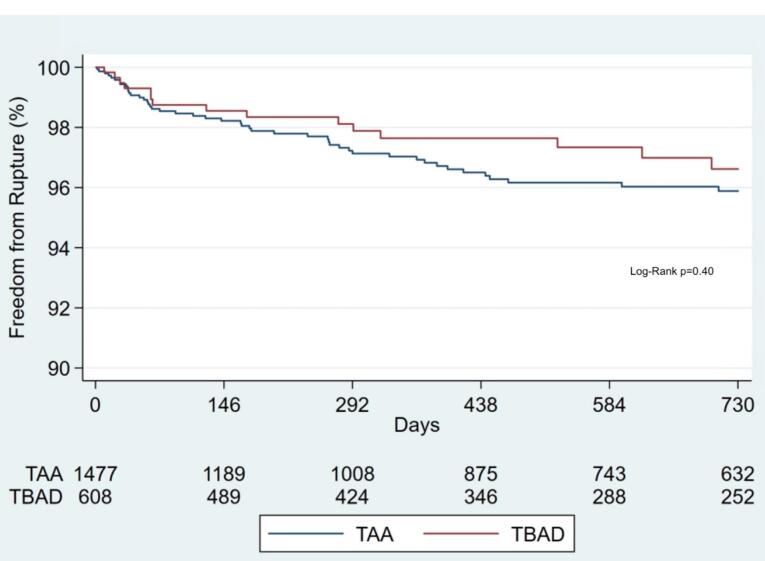
2-year Freedom from Rupture by Pathology

 There was no significant difference in all-cause mortality between TAA and TBAD, within the first year (20.1% vs. 17.4%, log-rank *P* = 0.09; Table 3, Figure 1) and at 2 years (29.0% vs. 24.7%, log-rank *P* = 0.07; Table 3, Figure 2). Within 1 year, patients with TBAD had a significantly higher incidence of aneurysm-related reintervention (18.8% vs. 12.9%, *P* = 0.003; Table 3, Figure 3). This finding persisted at 2 years (24.9% vs. 19.7%, *P* < 0.001; Table 3, Figure 4). There was no significant difference in rupture between the TAA and TBAD groups within 1 year (3.1% vs. 2.4%, *P* = 0.42; Table 3, Figure 5) and within 2 years (4.1% vs. 3.4%, *P* = 0.40; Table 3, Figure 6).

**Table 3:** Long-term outcomes following TEVAR for TBAD vs. TAA

**Outcome**	**TAA (n=1492, 70.9%)**	**TBAD (n=613, 29.1 %)**	**Log-Rank ** ***P*** ** value**	**aHR (95%CI) REF=TAA**	***P*** ** value**
All-cause mortality					
1-year	294 (20.1%)	100 (17.4%)	0.09	0.72(0.57-0.90)	0.004
2-year	378 (29.0)	129 (24.7%)	0.07	0.79(0.65-0.95)	0.013
Aneurysm-related reintervention					
1-year	161 (12.9%)	95 (18.8%)	0.003	1.44(1.03-2.04)	0.035
2-year	216 (19.7%)	114 (24.9%)	< 0.001	1.38(1.02-1.86)	0.037
Aneurysm rupture					
1-year	38 (3.1%)	12 (2.4%)	0.42	0.57(0.27-1.21)	0.14
2-year	47 (4.1%)	15 (3.4%)	0.40	0.57(0.29-1.14)	0.12

TAA: Thoracic aortic aneurysm, TBAD: Type B aortic dissection, OR: Odds ratio, CI: Confidence interval

 After adjusting for potential confounders, mortality was lower in the TBAD group compared to TAA at 1 year (aHR: 0.72, 95%CI: 0.57-0.90, *P* = 0.004) and also at 2 years (aHR: 0.79, 95%CI: 0.65-0.95, *P* = 0.013). Patients with TBAD had significantly higher risk of aneurysm-related reintervention at 1 year (aHR:1.44, 95%CI:1.03-2.04, *P* = 0.035) and at 2 years (aHR:1.38, 95%CI: 1.02-1.86, *P* = 0.037]) There was no significant difference in adjusted hazards of rupture at 1 year (aHR:0.57, 95%CI:0.27-1.21, *P* = 0.14) and 2 years (aHR:0.57, 95%CI: 0.29-1.14, *P* = 0.12).

## Discussion

 In this retrospective study of prospectively collected national data on the outcomes of TEVAR for TBAD versus TAA, while patients differed significantly in baseline characteristics, a comparable incidence of stroke, MI, and SCI was observed. However, the TBAD group had significantly lower mortality at 30 days, one, and two years. In addition, a higher risk of aneurysm-related reintervention at 1-year and at 2-years was observed in the TBAD group. Finally, there was no significant difference in mid-term rupture at 1-year and 2-years.

 Smoking is a known risk factor for both aortic aneurysms and dissections^12^, but a noteworthy finding was that 37% of TBAD patients were nonsmokers, in contrast to 21% of TAA patients. In addition, the TAA group was more likely to still be smoking within a month of surgery. These observations suggest that smoking may have a greater impact on the development of TAA.

 Some studies suggest a significant difference in 30-day mortality post-TEVAR for aneurysm vs. dissection^13,14^ while others report similar outcomes.^15,16^ Differences in early mortality observed in the TAA group can be attributed to underlying health conditions and older age. A recently developed risk predictor of 30-day mortality after TEVAR for TAA identified age ≥ 75, ASA class IV or V, CAD, procedural urgency, proximal landing zone < 3 and prior carotid revascularization as factors potentially associated with 30-day mortality.^17^ In our study, the TAA group was more likely to be older and have prior carotid revascularization. In addition, they were more likely to be on cardiovascular medications preoperatively, which likely signifies additional comorbidities, potentially placing the TAA group at a higher risk of 30-day mortality.

 Our findings are consistent with other studies that report similar stroke and SCI rates^15,18^ after TEVAR for aneurysm compared to dissection. In contrast to the aforementioned studies, the Waterford et al systematic review reported a significantly higher stroke rate in TAA vs. TBAD (4.3% vs. 3.2 %, *P* = 0.043) post-TEVAR.^19^ These results were obtained via analysis of 44 papers but only 12 of these papers directly compared post-TEVAR stroke rates between aneurysms and dissections. Whereas the other 32 papers examined post-TEVAR stroke rates in either aneurysms or dissections. This may explain the higher stroke rate seen in TAA patients.

 The co-existence of CAD in patients with aortic aneurysms, especially abdominal aneurysms, is common due to the presence of atherosclerosis, a known etiology of both pathologies.^20-22^ This may explain the higher prevalence of prior coronary revascularization and carotid interventions and the higher usage of pre-operative medications (aspirin, statin, and antiplatelet agents).

 Concomitant CAD and aortic aneurysms are associated with poor clinical outcomes after surgery.^23,24^ In patients undergoing endovascular and open repair for thoracic and abdominal aneurysms, Watanabe et al found that patients with non-significant CAD ( ≥ 25% but < 75% stenosis) and significant CAD ( ≥ 75% stenosis), when compared to the non-CAD group ( < 25% stenosis) had significantly more major adverse cardiovascular and cerebrovascular events (MACCEs).^25^ Specifically with TEVAR, the risk of postoperative MI is low (2.4%).^24^ This is similar to our finding of 2.4% in the TAA group and 1.3% in the TBAD group.

 Our findings are in agreement with older studies that have reported a significant difference in survival between descending thoracic aneurysms and type B dissections (1-year: 20.1% vs. 17.4%, *P* = 0.004, 2-years: 29.0% vs. 24.7%, *P* = 0.013.^14,16^ The reasonably acceptable post-TEAVR survival of approximately 80% at 1-year and 70% at 2-years suggests that TEVAR is well suited for the different pathologies. The higher midterm mortality observed with thoracic aneurysms has been attributed to associated comorbidities that these patients often present with.^14,16^

 The benefits of endovascular repair are often overshadowed by higher reintervention rates when compared to open repair.^26^ It has been demonstrated that aortic pathology type is a predictor of the need for reintervention.^14,27^ Prior studies have reported reintervention rates with TBAD following endovascular repair ranging from 12%-32%^26,28,29^ compared to a reintervention rate of 6.7%^27^ with TAA. In dissections, the graft is extended distally but often lands above the celiac artery. As a result, retrograde flow into the false lumen due to continued patency can occur which has been noted by numerous studies as a predictor of poorer outcomes and need for later reintervention.^30-32^ We observed a TBAD reintervention rate of 19% at 1 year and 25% at 2 years in our study. Compared to the reintervention rate reported at a similar follow-up period,^27^ the higher re-intervention rate reported in our study may be attributed to the higher risk patient population included in this study. Steuer et al reported a 35%^29^ rate of reintervention at five years, while Hanna et al reported a 24% reintervention rate at one year and a 31% reintervention rate at six years.^33^ Due to a smaller sample size of at-risk individuals, we were only able to assess reintervention at up to 2 years between the two group. However, one study looking at midterm outcomes following TEVAR reported a significantly higher reintervention rate in patients with acute TBAD and chronic TBAD compared to TAA patients at six years.^14^ The higher rate of reintervention following TEVAR for both aneurysms and TBAD highlights the importance of continued surveillance after surgery.

 Another noteworthy finding was the rates of post-TEVAR rupture at 2 years (TAA = 4.1% vs. TBAD = 3.4%, *P* = 0.40). This is in comparison to a reported yearly post-EVAR rupture rate ranging from 0.5%-1.2%.^34^ A recent series examining 10-year rupture in patients undergoing open abdominal aortic aneurysm repair versus endovascular abdominal aortic repair reported a rupture rate of 6.6% vs. 10.3%, *P* < 0.001 respectively.^35^ It is therefore important to emphasize the importance of close follow-up after the index procedure. This study has several limitations. First, it is a retrospective which precludes causal inferences and is subject to inclusion bias. Nevertheless, retrospective studies play an important role in addressing important clinical questions in surgery and can serve as a complement to randomized controlled trials.^36^ Secondly, inherent to retrospective observational studies is the lack of randomization, which subjects the analysis to potential confounders. However, we attempted to adjust for all measurable confounders reported in this database. Third, the presence of unmeasured confounders not captured in this dataset may influence outcomes. Fourth, since our population was derived from self-reported data, this may introduce missing data and human error. However, this data is subject to regress auditing and comparison to claim data. Fifth, mortality in this study was defined as all-cause mortality, and we were not able to specifically analyze TEVAR-related mortality, which may limit interpretation of outcomes directly attributable to the procedure. Finally, due to the smaller sample size of at-risk individuals after year two, we were unable examine long-term outcomes, limiting the generalizability of our results beyond two years. Future studies with longer follow-up periods are needed to better assess changes in mortality, reintervention and rupture over time.

## Conclusion

 In this large multi-institutional national study, patients undergoing TEVAR for TAA and TBAD differed in terms of baseline characteristics and postoperative and long-term outcomes. Patients undergoing TEVAR for TBAD had a 38% increased risk of reintervention at two years compared to patients with TAA. The midterm-all-cause mortality reported in this analysis suggests worse outcomes for TAA, though with similar rupture rates. However, with up to 4% post-TEVAR rupture, close follow-up is necessary to identify patients who may require reintervention to maintain the integrity of the repair.

## Competing Interests

 None

## Ethical Approval

 No need for IRB approval/exemption since this is de-identified data.
